# Technical note: CT-guided biopsy of lung masses using an automated guiding apparatus

**DOI:** 10.4103/0971-3026.54883

**Published:** 2009-08

**Authors:** Amarnath Chellathurai, Saneej Kanhirat, Kabilan Chokkappan, Thiruchendur S Swaminathan, Nadhamuni Kulasekaran

**Affiliations:** Barnard Institute of Radiology, Madras Medical College, Government General Hospital, Chennai - 600 003, India

**Keywords:** Automated / manual planning, CT-guided needle lung biopsy

## Abstract

Automated guiding apparatuses for CT-guided biopsies are now available. We report our experience with an indigenous system to guide lung biopsies. This system gave results similar to those with the manual technique. Automated planning also appears to be technically easier, it requires fewer number of needle passes, consumes less time, and requires fewer number of check scans.

## Introduction

CT-guided lung biopsy is a usually done manually, using a standard technique. For some years now, automated systems have been available to guide biopsies.[1,2] We discuss our experience with a newly developed indigenous system.

## Technique

**Figure 1:**
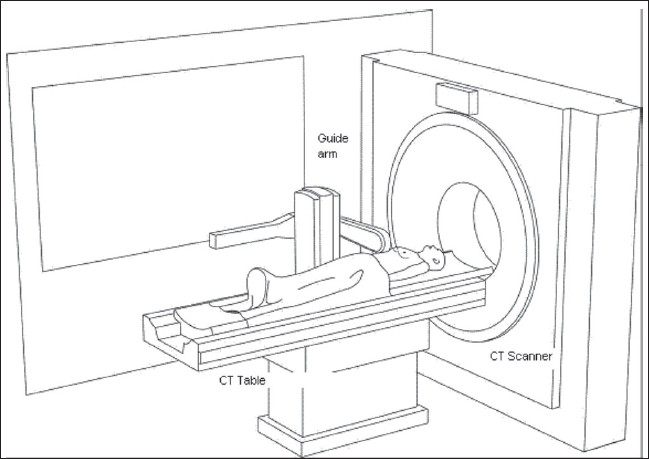
The apparatus (arrow) is a stand-alone mobile system that works in conjunction with a CT system. The apparatus is locked, with respect to the CT system, through a docking plate fixed to the floor and to the device

We used PIGA-CT (a robotic five-axes guide arm and planning console) designed by Perfint Healthcare Pvt. Ltd. (Chennai, India); it is an automated apparatus that calculates coordinates on DICOM images from a CT scanner and guides the placement of a needle accurately within the body after insertion [Figure 1].

**Figure 2:**
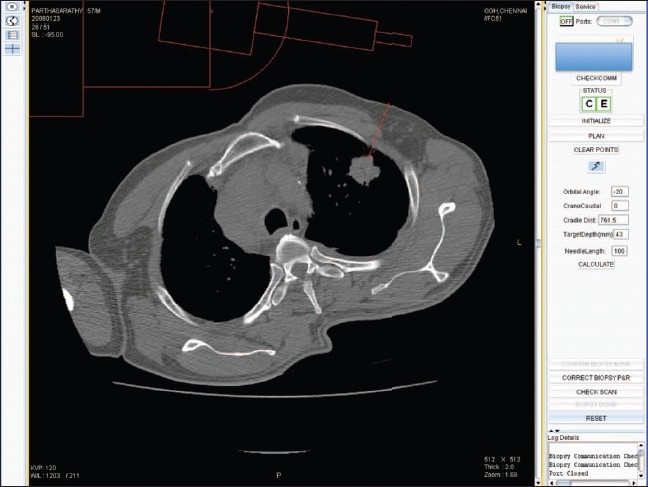
The radiologist selects the appropriate slice(s) wherein the needle has to enter. Once the desired point of entry and the target are marked on the console (called plan), the coordinate information is sent to the guide arm

**Figure 3:**
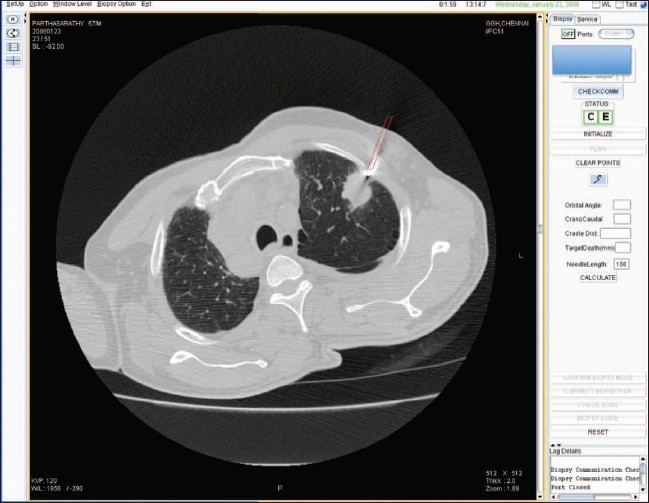
The cradle is automatically moved to the position indicated by the software; the arm is positioned over the patient in such a way that only the required length of the needle (arrow) is inserted, reducing the risk of overshooting of the needle

The apparatus consists of an electromechanical guide arm that provides five degrees of freedom, a computer console for receiving CT images and calculating coordinates, and an RS232 interface for data communication between the guide arm and the computer console. Precise ‘point of insertion’ and ‘point of target’ are determined from the images and marked. The apparatus is able to accurately position itself by using the movement in five axes. The manipulator aligns the needle guide. The needle is required to enter the body at the ‘point of insertion’ and to touch the target at the ‘point of target’ [Figures 2 and 3].

Of 36 consecutive CT-guided needle biopsies of the chest performed at our institute between 30^th^ June 2007 and 24^th^ January 2008, 18 (group I) were performed using manual planning and 18 (group II) with the automated biopsy system. A four-slice CT scanner (Toshiba; DICOM compatible) was used to localize the lesion and to guide needle placement. No cytopathologists were present at the time of biopsy.

Seven (19%) small post-biopsy pneumothoraces complicated the 36 procedures; none of them required placement of a chest tube. Four cases occurred while using the manual method and three during the automated method. In this study, the technical success was 100% with both the methods. Using the manual method, 11 biopsies (61.1%) yielded sufficient tissue for pathologic evaluation whereas, with the automated apparatus, 12 (66.7%) biopsies gave a definitive diagnosis.

## Discussion

We were able to show that the automated system works well and could provide technical and diagnostic success rates similar to those obtained with the manual method. Also, we found that the automated device decreased the number of needle position adjustments and thereby minimized the procedure time. There was no significant difference in the incidence of complications with the two methods.

In our opinion, such automated systems can be extremely useful when the radiologist doing the biopsy has limited experience or when the lesion is situated in difficult locations. Larger trials are required to assess the usefulness and cost-effectiveness of such automated systems in different clinical environments.
